# Effect of Mesoporous Silica Nanoparticles on The Physicochemical Properties of Pectin Packaging Material for Strawberry Wrapping

**DOI:** 10.3390/nano10010052

**Published:** 2019-12-24

**Authors:** Asmaa Al-Asmar, C. Valeria L. Giosafatto, Mohammed Sabbah, Alfredo Sanchez, Reynaldo Villalonga Santana, Loredana Mariniello

**Affiliations:** 1 Department of Chemical Sciences, University of Naples “Federico II”, 80126 Naples, Italy; asmaa.alasmar@unina.it (A.A.-A.); giosafat@unina.it (C.V.L.G.); 2 Analysis, Poison Control and Calibration Center (APCC), An-Najah National University, P.O. Box 7, Nablus, Palestine; 3 Department of Nutrition and Food Technology, An-Najah National University, P.O. Box 7, Nablus, Palestine; m.sabbah@najah.edu; 4 Nanosensors and Nanomachines Group, Department of Analytical Chemistry, Faculty of Chemistry, Complutense University of Madrid, 28040 Madrid, Spain; alfredos@ucm.es (A.S.); rvillalonga@quim.ucm.es (R.V.S.)

**Keywords:** pectin, mesoporous silica nanoparticles, wrapped strawberries, food packaging, biodegradable films

## Abstract

Citrus peel pectin was used to prepare films (cast with or without glycerol) containing mesoporous silica nanoparticles. Nanoparticles reduced significantly the particle size, and had no effect on the Zeta potential of pectin solutions. Mechanical characterization demonstrates that pectin+nanoparticles containing films slightly increased tensile strength and significantly decreased the Young’s modulus in comparison to films made only of pectin. However, elongation at the break increased in the pectin+nanoparticles films cast in the presence of glycerol, while both Young’s modulus and tensile strength were reduced. Moreover, nanoparticles were able to reduce the barrier properties of pectin films prepared with or without glycerol, whereas positively affected the thermal stability of pectin films and the seal strength. The 0.6% pectin films reinforced or not with 3% nanoparticles in the presence of 30% glycerol were used to wrap strawberries in order to extend the fruit’s shelf-life, over a period of eighty days, by improving their physicochemical properties.

## 1. Introduction

Nowadays, people are more aware about the harmful effects of the presence in the environment of wastes derived from plastic materials [1]. Globally, production of plastics exceeds 300 million tons per year, and it is likely that a similar quantity of plastics will be produced in the next eight years as it was produced during the twentieth century [2]. A possible solution to reduce the consumption of traditional plastics is to produce innovative biomaterials with promising properties. Hydrocolloids are the main macromolecules naturally available to produce innovative products suitable in many sectors, i.e., food, agriculture, bioplastic and pharmaceutical industries [3]. Therefore, the development of potential hydrocolloid-based coatings and packaging biopolymers has increased during the last years. 

However, some properties of these kinds of films are, so far, still poor, and further studies are necessary to find new additives to be added into biomaterials with improved both mechanical and barrier characteristics, both mechanical and barrier to gases and water vapor [4,5].

Pectin (PEC) is a complex anionic polysaccharide mainly composed of homogalacturonan (1 → 4 linked α-_D_-galacturonic acid and its methyl ester) [6]. PEC has been used in the food and beverage industries for many years. The principal applications of PEC are as a gelling agent, thickening agent, stabilizer and emulsifier [7,8]. Recently, Al-Asmar et al. [9] demonstrated that the PEC-based coating was the most effective among other hydrocolloid-based coatings (containing chitosan or proteins) in reducing acrylamide formation during the frying of French fries. However, as PEC-based films have some limitations regarding the mechanical properties, so many researchers have studied how to improve them by adding proteins [10,11,12], and/or new plasticizers [13], in order to improve flexibility and to reduce the rigidity and brittleness of the films. 

The effect of active compounds inside the film generally depends on the physicochemical properties of the matrix structure, which in turn depends on its morphology at the nanoscale level [14]. For these reasons, many different studies are being devoted to film reinforcement by nanomaterials able to act also as nanocontainers for active compounds [14,15,16,17,18]. More recently, the use of nanoparticles in the development of nanocomposite materials is becoming to represent a new strategy also to improve the physical properties of several polysaccharide-based materials, and the food industry might benefit from it mainly through the production of innovative active and intelligent packaging [19]. Mesoporous silica nanoparticles (MSNs), one of the most important porous materials, have been recently widely used due to their unique features, such as high surface area, controllable pore structure, large pore volume, optically transparent properties, low toxicity, high chemical and thermal stability and a versatile chemical modifiable surface [20,21,22,23]. Recently, Fernandez-Bats et al. [14] prepared and characterized the active protein edible films nanostructured with MSNs or with their amino-functionalized derivative, and they concluded that the film tensile strength and elongation at break significantly increased in the presence of both kinds of nanoparticles. Moreover, Tyagi et al. [24], demonstrated that the incorporation of silica with polysaccharide has a significant reduction to the rate of water vapor transmission and water vapor permeability of the film. However, amorphous hybrids materials with the participation of pectin and silica derived from aqueous silicates and tetraethoxysilane (TEOS) were successfully prepared [25,26]. 

The possible health risk of the consumption of food containing nanoscale compounds transferred from the packaging is not yet fully studied. So far, few studies have reported that the safety of food-containing nanoparticles depends upon the particles’ toxicity, size, morphology and the rates of migration and ingestion [27].

The strawberry (*Fragaria vesca*) is one of the most perishable fruits, and has very short postharvest time, because of its susceptibility to mechanical damage, physiological deterioration and possible attack of pathogens [28]. However, strawberry is classified as a healthy food, due to a high content of vitamin C, antioxidant activity, vitamin E and phenolic compounds which make *Fragaria vesca* important for human nutrition [29,30,31]. To date, some studies have been devoted to extend the shelf life of strawberry fruits, for example, by dipping fruits with edible coatings [32,33], by packaging using chitosan–poly(vinylalcohol) (PVA) blend films [34], and by using ϒ-irradiation [35].

The objective of this study was to investigate the effect of nanoreinforcement on both PEC films-forming solutions (FFSs) and cast film physiochemical properties. Samples, prepared at two different concentrations of PEC, glycerol (GLY) and/or MSNs, were prepared with the aim of obtaining a suitable biomaterial useful for food packaging. We have here tested the efficiency of PEC-based films in protecting strawberries with the aim of extending their shelf-life at 4 °C.

## 2. Materials and Methods

### 2.1. Materials

Citrus peel low-methylated (7%) PEC (Aglupectin USP) was obtained from Silva Extracts srl (Gorle, BG, Milan, Italy), while glycerol (GLY) (about 87%) was purchased from the Merck Chemical Company (Darmstadt, Germany). Tetraethylortosilicate (98%) (TEOS) and cetyltrimethylammonium bromide (CTAB) were obtained from Sigma (Steinheim, Germany) that was used to synthesize the mesoporous silica nanoparticles (MSNs), as recently reported by Fernandez-Bats et al. [14]. MSN-based solutions were prepared with distilled water [14]. 2,2-diphenyl-1-picrylhydrazyl (DPPH) was purchased from Sigma Chemical Company (Pool, Dorset, UK), while ascorbic acid was from Merck Chemical Company (Darmstadt, Germany). Other chemicals and solvents used in this study were of analytical grade. Mater-Bi^®^ (S 301)-based commercial material bags were from a local market, Naples, Italy. The strawberry variety “Sabrina” was purchased from a local market in Naples, Italy, and strawberry experiments were carried out the day after their purchasing.

### 2.2. PEC Film Forming Solutions (FFSs) and Film Preparation

A stock solution of PEC (2.0 g) was dissolved in 100 mL of distilled water until the PEC solution was completely solubilized. Serial concentrations of PEC-based films-forming solutions (FFSs) were prepared at pH 7.5 from 0.2–1%, containing MSN 3% *w*/*w* PEC in the absence and presence of GLY (30% *w*/*w* PEC). Then the 6 and 10 mg/mL PEC FFSs with different concentrations of MSNs (1%, 3% and 5% *w*/*w* PEC), were prepared both in the absence and presence of different concentrations of GLY (10, 30 and 50% *w*/*w* PEC) in 50 mL of distilled water. The same volumes (50 mL) of all the different FFSs, containing or not MSNs and/or GLY, were cast onto 8 cm diameter polystyrene Petri dishes (6 and 10 mg PEC/cm^2^, respectively) and finally the films were allowed to dry in an environmental chamber at 25 °C and 45% RH for 48 h. The handleable dried films were peeled off intact from the casting surface after they were conditioned at 25 °C and 53% RH for 2 h in a desiccator containing a saturated solution of Mg(NO_3_)_2_·6H_2_O.

### 2.3. Zeta Potential and Particle Size Measurements

Zeta potential and mean particle size hydrodynamic diameter (Z-average size) of the 1 mg/mL PEC FFSs prepared at pH 12.0, containing or not MSN 3%, GLY 30% or both (*w*/*w* PEC), were titrated automatically from pH 12.0 to pH 2.0, by measuring the dynamic light scattering by a Zetasizer Nano-ZSP (Malvern^®^, Worcestershire, UK) using a He–Ne laser (wavelength of 633 nm) and a detector angle of 173°. 

### 2.4. Film Thickness 

Film thickness was measured with an electronic digital micrometer (DC-516, sensitivity 0.001 mm) at different positions of each film sample. At least six measurements were taken on each film sample, and the thickness mean values were considered in the different tests.

### 2.5. Film Mechanical Properties

The mechanical properties tensile strength (TS), elongation at break (EB) and Young’s modulus (YM), were measured according to ASTM D882 [36], by using a universal testing instrument model no. 5543A (Instron, Norwood, MA, USA). PEC films strips (1 cm wide and 5 cm long) were obtained by using a sharp scissors, and were conditioned in an environmental chamber at 25 °C and 53% RH for 2 h. Finally, six samples of each film type were tested, and the speed was 5 mm/min in tension mode.

### 2.6. Seal Strength 

The seal strength of each of the PEC films was tested by the Instron universal testing instrument model no. 5543A (Instron Engineering Corp., Norwood, MA, USA). PEC film samples were cut into strips of 5 × 2.5 cm, and one strip was placed on the top of another. After being sealed, film samples were conditioned at 25 °C and 50% ± 5% RH for 48 h [37,38,39], and few drops of water were added to the seal area. Those two strips were sealed by a heat sealer (MAGIC VAC^®^ AXOLUTE Mod: P0608ED). The width of the seal area was 0.3 cm^2^. The sealing parameters were studied according to ASTM F 88-07a (2007) [40], the seal strength (N/m) was calculated as following Equation (1) [37,39]:

Seal strength (N/m) = Peak force/film width (1)

### 2.7. Film Barrier Properties 

The gas permeability tests toward O_2_ [41], CO_2_ [42], and water vapor (WV) permeability [43] of triplicate samples of each film were performed at 25 °C under 50% RH, in aluminum masks having an area of 5 cm^2^, by using MultiPerm apparatus (ExtraSolution s.r.l., Pisa, Italy).

### 2.8. Differential Scanning Calorimetry (DSC) and Thermogravimetric Analysis (TGA)

Differential Scanning Calorimetry (DSC) and Thermogravimetric Analysis (TGA) of the films were performed on a DSC Q-200 (TA Instruments, New Castle, United States). Samples (about 10 mg) were placed into platinum crucibles and then they were heated at 5 °C/min, from room temperature to 400 °C, under inert atmosphere (50 mL/min of N_2_).

### 2.9. Strawberry Experiments 

#### 2.9.1. Strawberry Wrapping

The selected strawberries were of uniform size, color and without physical damages and fungal infections. They were randomly divided into four groups. Each group of eleven strawberries was wrapped (W) by different sealed films (10 cm × 10 cm) as following: 0.6% PEC + 30% GLY film (W, PEC+GLY), 0.6% PEC + 3% MSN + 30% GLY film (W, PEC+MSN+GLY), Mater-Bi^®^ commercial material (W, Mater-Bi) and the control was unwrapped (UW). These samples were placed at 4 °C, the quality of both wrapped and control samples were evaluated during storage at 0, 2, 4, 6 and 8 days.

#### 2.9.2. Weight Loss

The weight loss of the unwrapped and wrapped samples was calculated in triplicate at 0, 2, 4, 6 and 8 days of storage as following Equation (2) [34]:

Weight loss (%) = ((W_0_ − W_1_)/W_0_) × 100 (2)

where W_0_ and W_1_ represent initial and final fruit weights, respectively.

#### 2.9.3. Determination of pH and Titratable Acidity

Five fruits were taken from each group and then 5 g of fresh homogenate was suspended in 50 mL distilled water using a blender and then centrifuged at 5000 rpm for 10 min. Titratable acidity (TA as citric acid %, using 0.064 as conversion factor for citric acid), was calculated by titrating 5 mL of clear strawberry juice diluted in 50 mL of deionized water against 0.1 N NaOH solution [44], and then the pH of the samples was measured by a digital pH meter. All sample determinations were in triplicate during storage at 0, 2, 4, 6 and 8 days at 4 °C.

#### 2.9.4. Ascorbic Acid Content and DPPH Radical Scavenging Activity

Ascorbic acid was determined using DPPH as Equivalent Antioxidant Capacity according to the procedure previously described by Almeida et al.; Brand-Williams et al. and Giosafatto et al. [45,46,47], with some modifications: the solution of DPPH (0.05 mg/mL) was diluted with methanol in order to obtain an absorbance of 1.516 ± 0.04 at 517 nm. Homogenous strawberry fruit (100 μL) or controls ascorbic acid were allowed to react with (900 μL) of DPPH radical solution for 30 min in the dark, and the decrease in absorbance from the resulting solution was adjusted. The standard curve of 0–80 mg of ascorbic acid/100 mL was linear (y = −0.017x + 1.484, R^2^ = 0.992). 

The same sample were used to study the DPPH radical scavenging activity assay as antioxidant activity %, based on the method previously described by Giosafatto et al. and Odriozola-Serrano et al. [47,48]. The absorbance was measured in triplicate as fresh weight at 517 nm for all samples. Results were expressed (antioxidant activity %) as following Equation (3) [47]:

Antioxidant activity % = ((Abs DPPH − Abs sample)/Abs DPPH) × 100(3)

#### 2.9.5. Texture Profile Analysis (TPA)

The Texture Profile Analysis (TPA) of unwrapped and wrapped samples stored for 0, 4 and 8 days was performed as described by [49,50]. Texture analyses were carried out using an Instron universal testing instrument model no. 5543A (Instron Engineering Corp., Norwood, MA, USA) equipped with a 2 kN load cell in compression mode with a cylindrical probe (55 mm in diameter) [50]. The test was configured so that the TPA parameters: (a) Hardness (N), defined as maximum force of the first compression peak; (b) chewiness (N.mm), defined as the total amount of work necessary to chew a sample until it is ready for swallowing [50]; (c) gumminess (N), calculated automatically by multiplying the hardness with the ratio of the positive force areas under first and second compressions (cohesiveness). All TPA parameters were calculated by the software Bluehill by determining the load and displacement at predetermined points on the TPA curve. Pre- and post-test speeds were 2.0 mm/s, while the test speed was 1.0 mm/s. The strawberries were centered and compressed to 60%, and 20 mm of its original size of deformation, and then the average hardness, chewiness and gumminess values of at least six strawberries of each group were evaluated.

### 2.10. Statistical Analysis

John’s Macintosh Project (JMP) software 5.0 (SAS Institute, Cary, NC, USA) was used for all statistical analyses. The data were subjected to analysis of variance (ANOVA), and the means were compared using the Tukey–Kramer honestly significant difference (HSD) test. Differences were considered to be significant at *p* < 0.05.

## 3. Results and Discussion

### 3.1. Zeta Potential and Particles Size of PEC and MSN-Based Solutions

**Figure 1:**
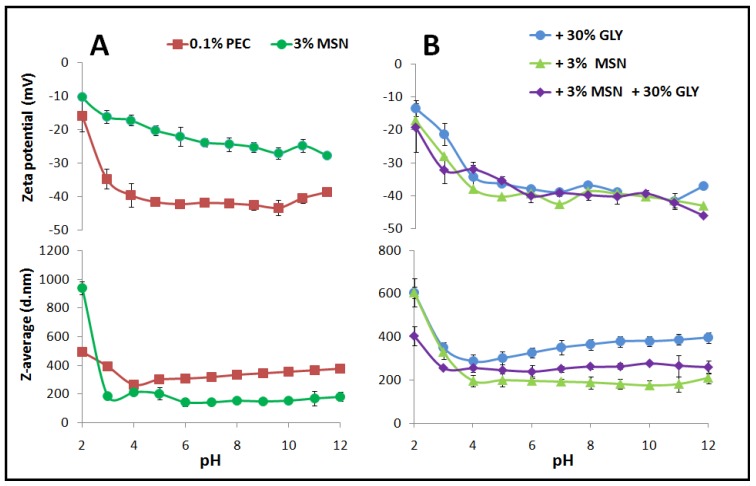
Effect of different pHs on the Zeta potential and Z-average size of 0.1% PEC-based and 3% MSN water solution (Panel **A**), and of 0.1% PEC-based FFS (prepared in the presence of 30% GLY (*w*/*w* with PEC) or 3% MSNs (*w*/*w* with PEC) or in the presence of both 3% MSNs and 30% GLY (Panel **B**).

In order to verify the effect of pH on the stability and particles size of PEC-based FFS and MSN-based solutions, automatically, titrations were performed starting from pH 12 to pH 2. Figure 1 (upper part of Panel A) shows that the Zeta potential of 0.1% PEC-based FFS is highly stable, being around −40 mV, starting form pH 12 to ≈ pH 4. These results are in good agreement with those previously obtained on the PEC-based films [13,51]. Moreover, the Z-average was ≈ 300 d.nm in the same pH range (Figure 1, lower part of Panel A). However, the 3% MSN-based solutions, rich in OH groups, show less stability compared to PEC-based FFS, since the Z-potential range was ≈ −28 mV to −20 mV from pH 12 to pH 5, respectively, reaching −10 mV at pH 2 (Figure 1, upper part of Panel A). These results were in agreement with those obtained by Fernandez-Bats et al. [14]. Regarding the Z-average, MSNs showed a smaller size of about 150 d.nm, at pH values ranging from 12 to 6 (Figure 1, lower part of panel A). 

This value was very similar to the average size of MSN-based nanoparticles obtained by Fernandez-Bats et al. and Musso et al. [14,52]. The latter authors have characterized MSNs also by TEM, observing an average size of 143 ± 26 nm. At pH 3, the MSN particle size reached about 200 d.nm. 

In general, PEC films present some limitations such as rigidity and brittleness [53]. In this work, GLY has been used as a plasticizer to improve PEC-based formulation properties by adding GLY or MSNs, or both. In particular, Figure 1, Panel B, reports the influence of pH on Zeta potential and Z-average size of 0.1% PEC FFSs containing 30% GLY, or 3% MSNs or both MSNs and GLY. The results show that, adding either GLY or MSNs does not change the PEC FFS stability from pH 12 to pH 6 (Figure 1, upper part of Panel B). Regarding the Z-average, Figure 1 (lower part of Panel B) shows that the presence of both MSNs and GLY into the PEC-based solution provokes a reduction of particle size from 600 to 400 nm at pH 2, reaching a diameter of around 200 nm at pH ≥ 3. To study the effect of different concentrations of each component, pH 7.5 was selected to cast the films in the further studies.

### 3.2. Mechanical Properties 

**Figure 2:**
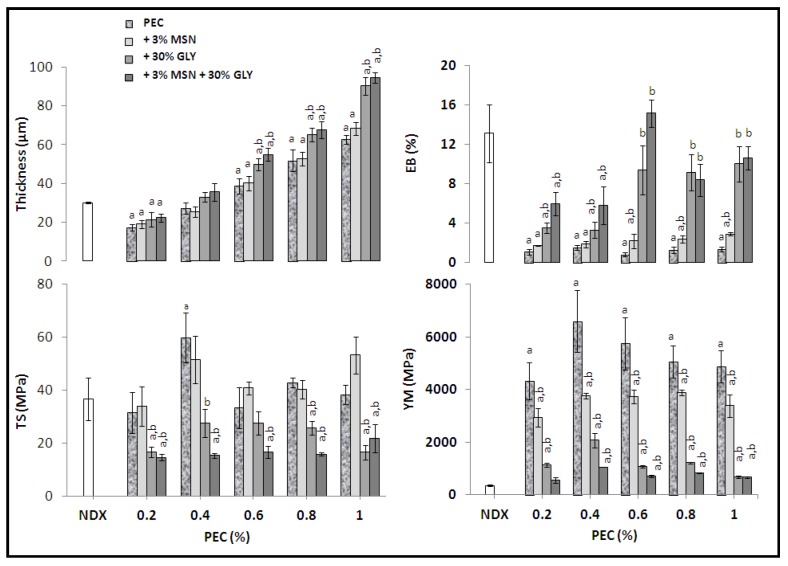
Effect of pectin (PEC) concentration on thickness and mechanical properties of films prepared at pH 7.5 in the presence or absence of mesoporous silica nanoparticles (MSNs) or glycerol (GLY) or in the presence of both. The values, significantly different from those obtained by analyzing the Viscofan NDX edible film (white bars) (Data from Porta et al. [54]), are indicated by “a”, while the values indicated by “b” were significantly different from those obtained by using only PEC (*p* < 0.05). Further experimental details are given in the text.

Figure 2 reports the film thickness and mechanical properties of PEC-based films prepared with different concentrations of PEC (0.2%, 0.4%, 0.6%, 0.8% and 1%) either in the presence or absence of 3% MSNs or 30% GLY and in the presence of both. The results were compared with the Viscofan NDX edible film that is a commercially available material prepared from collagen and mainly used in the meat industry [54]. 

The results indicate that film thickness increases depending on PEC concentration, becoming thicker, then also NDX at 0.4% and higher concentrations. MSNs do not affect PEC film thickness. Moreover, GLY influences thickness significantly when added alone or together with MSNs in films prepared at PEC concentrations equal or superior to 0.6%. PEC concentration seems do not affect EB that is very low in PEC films compared to NDX material. The presence of GLY and the concomitant presence of both GLY and MSNs influence EB significantly when 0.4% and higher PEC concentrations were used. 

Giosafatto et al. [55], evaluated the effect of 3% MSNs or/and 30% GLY on the 0.6% PEC and 0.6% chitosan, and they demonstrated that MSNs and GLY significantly increased the plasticity of both kinds of films. In particular, the PEC-based films containing MSNs or GLY showed a higher value of EB, and a reduced YM, while a more marked effect on these parameters was obtained by adding both MSNs and GLY. At the same time, the concurrent presence of MSNs and GLY significantly reduced the tensile strength of the PEC-based films [55].

It can be of interest to recall that Fernandez-Bats et al. [14] have demonstrated that protein-based films reinforced with MSNs showed higher TS and EB, reaching the highest values using 3% MSNs. Nesic et al. [56], studying highly methylated pectin biocomposite membranes reinforced by MSN type SBA-15, observed an increase of both TS and EB, but a reduction in YM.

**Figure 3:**
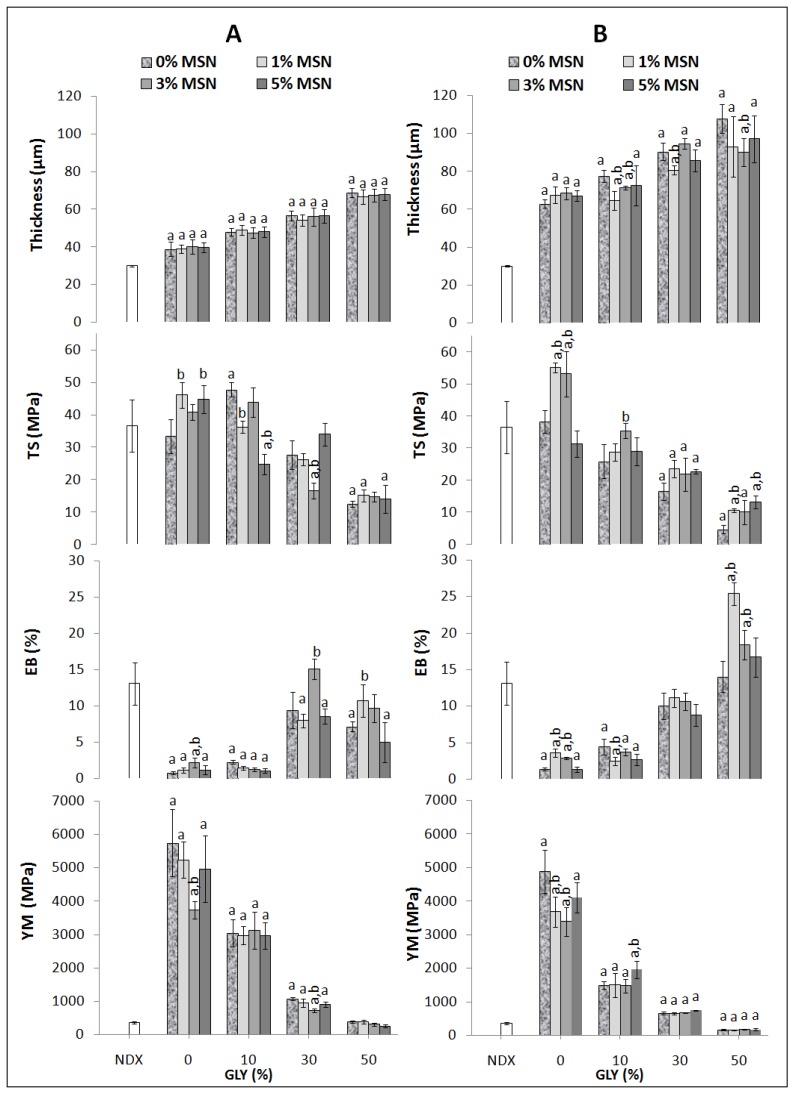
Effect of different concentrations of MSNs and GLY on thickness and mechanical properties of 0.6% PEC films (Panel **A**) and 1% PEC films (Panel **B**), both prepared at pH 7.5. The values significantly different from those obtained by analyzing Viscofan NDX (white bars) (Data from Porta et al. [54]), are indicated by “a”, while the values indicated by “b” were significantly different from those obtained by analyzing films made of either only PEC or the one obtained with GLY (*p* < 0.05). Further experimental details are given in the text.

For further studying the influence of both MSNs and GLY on film thickness and mechanical properties, 0.6% and 1% PEC-based films were chosen. Figure 3 shows that, in both kinds of films, thickness increases significantly by increasing the GLY concentrations, using 10%, 30%, or 50% of the plasticizer, respectively. No effect was observed on the 0.6% PEC film thickness by changing the MSN concentrations. Regarding 1% PEC films, the thickness was significantly reduced when the films were prepared in the presence of 10% GLY with both 1% and 3% MSNs. Thickness of films made in the presence of 30% GLY was affected only with 1% MSNs, while thickness of films made in the presence of 50% GLY was affected when 3% MSNs were added. Moreover, the presence of either 1% or 3% MSNs in FFS containing 50% GLY provokes an increased EB compared to the NDX material and to all other film kinds. However, 50% GLY-containing films showed lower TS and YM among all the studied films. 

In summary, regarding the mechanical properties the GLY component is the one that affects TS (a measure of resistance), since it reduces this parameter, while it increases EB (a measure of extensibility) and decreases the YM (a measure of stiffness). On the contrary, MSNs influence positively the resistance and the extensibility, while they also contribute to obtain a more rigid material. Taking into account the film characterization results (thickness, mechanical and permeability properties), an efficient wrapping bioplastic could be obtained made of 0.6% PEC, 3% MSNs and 30% GLY.

### 3.3. Heat Seal Strength

**Figure 4:**
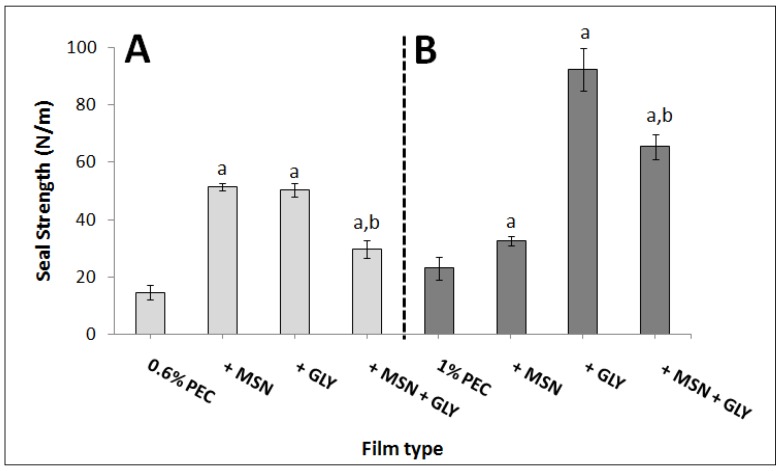
Seal strength of 0.6% PEC film prepared with or without 3% MSNs (*w*/*w* PEC), 30% GLY (*w*/*w* PEC) or in the presence of both (Panel **A**) and 1% PEC film prepared with or without 1% MSN (*w*/*w* PEC), 50% GLY (*w*/*w* PEC) or in the presence of both (Panel **B**). All the films were prepared at pH 7.5. The values significantly different from those obtained by PEC only (0.6% or 1%) are indicated by “a”, while the values indicated by “b” were significantly different from those obtained by only adding GLY (*p* < 0.05).

An important parameter for bioplastics, that have to be used for wrapping, is the heat seal strength. A higher heat seal strength value indicates that a higher strength is necessary to separate two parts of the same material that have been sealed to each other. Our findings indicated that the presence of either MSNs or GLY in PEC-based films increased the seal strength significantly. The 1% PEC-based films gave higher values compared to 0.6% PEC-films (Figure 4). However, the incorporation of both MSNs and GLY significantly reduced the seal strength in comparison to the values obtained with PEC+GLY. 

In other words, MSNs counteract the sealability of the PEC+GLY-based films. Similar results were also reported by Voon et al. [18], since they found out that using both nanoparticle halloysite nanoclay and SiO_2_ reduced the seal strength of bovine gelatin-based film plasticized with 20% GLY. However, increasing PEC (from 0.6% to 1%) and GLY (from 30% to 50%) concentrations improved the seal strength (Figure 4). This behavior was also observed by Farhan and Hani [37], that obtained an increase of the seal strength by increasing the plasticizer (GLY or sorbitol) concentrations in kappa-carrageenan films. These results could be likely due to higher heat transfer between the two sealed surfaces [37,57]. Generally, a higher value of seal strength is desirable when a higher capacity to resist the separation is required, whereas a lower value of seal strength might be advantageous in some applications where an easy peel opening is needed. For example, in the food field, consumers may prefer uncomplicated packaging to be open [18].

### 3.4. CO_2_, O_2_, and Water Vapor (WV) Permeability

Table 1 reported the effect of MSNs and/or GLY on gas (CO_2_ and O_2_) and the WV permeabilities of the PEC-based films. The permeability of 0.6% PEC film toward the CO_2_ and O_2_ were decreased significantly by adding 3% MSNs or 30% GLY or both. 

However, adding 1% MSNs to 1% PEC-containing films does not decrease the CO_2_ permeability, while it does reduce significantly the O_2_ permeability. On the other hand, the only addition of 50% GLY to 1% PEC film increases significantly the permeability to both gases, while it decreases significantly when 1% of the MSNs were added to 1% PEC film prepared in the presence of 50% GLY.

The strong effects of MSNs were markedly indicated by reducing significantly the WV permeability of both 0.6% and 1% PEC films obtained in the presence or in the absence of GLY. The gases and WV permeability values of MSN-reinforced 0.6% PEC film in the presence of GLY were lower compared to other polysaccharides like chitosan and commercial materials such as Viscofan NDX or Mater-Bi^®^. This result was obtained also by Fernandez-Bats et al. [14], that have studied protein films, concluding that the MSN addition gives rise to materials less permeable to gases and WV. The obtained results could be explained taking into account the structure of such films studied in a recent work [55]. In fact, in this study it has been reported that MSN-containing PEC-based films possess a more compact and homogeneous structure in comparison to the film prepared without any MSNs [55].

### 3.5. Thermal Properties 

**Figure 5:**
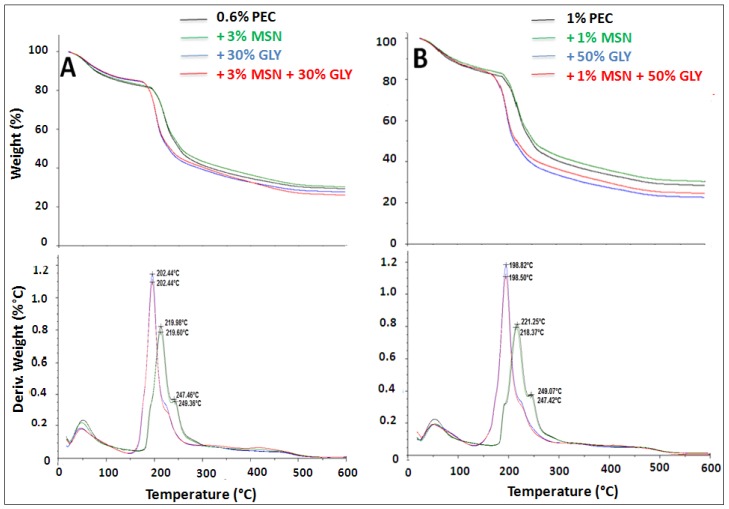
Thermogravimetric analysis (TGA) and differential thermal gravimetry (DTG) of 0.6% PEC film prepared at pH 7.5 with or without 30% (*w*/*w* PEC) GLY, 3% MSNs (*w*/*w* PEC) or with both (Panel **A**) and 1% PEC film prepared with or without 50% (*w*/*w* PEC) GLY, 1% MSNs (*w*/*w* PEC) or with both (Panel **B**).

Figure 5 shows the TGA and differential thermal gravimetry (DTG) curves of 0.6% (Panel A) and 1% (Panel B) PEC films prepared with MSNs or GLY or with both. The weight loss curves of all samples exhibited a first weight loss appearing from room temperature to about 110 °C, representing evaporation of water molecules, present mainly as free water [59]. 

**Figure 6:**
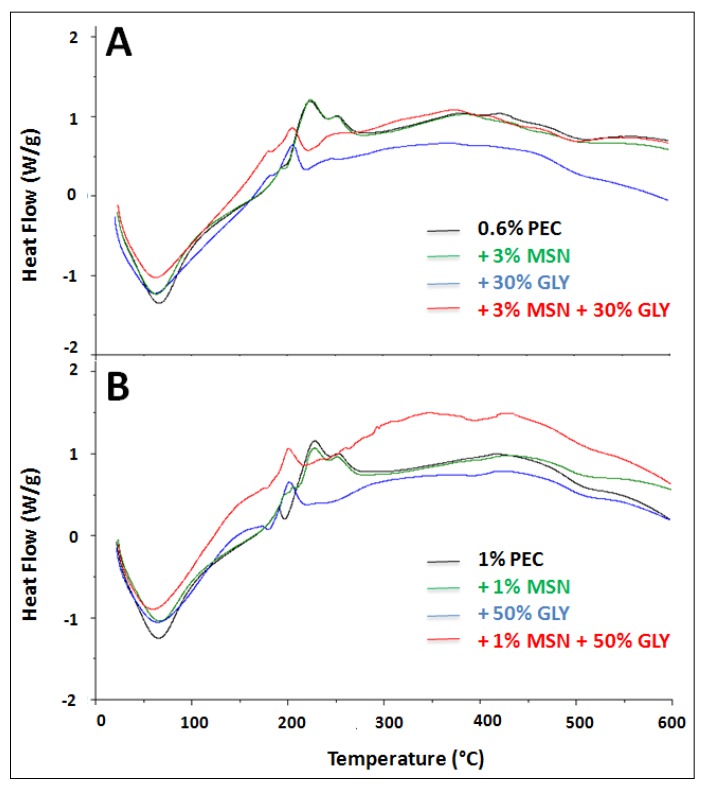
Differential Scanning Calorimetry (DSC) thermograms of 0.6% PEC film prepared with or without 30% GLY (*w*/*w* PEC), 3% MSNs (*w*/*w* PEC) or in the presence of both (Panel **A**) and of 1% PEC film prepared with or without 50% GLY (*w*/*w* PEC), 1% MSNs (*w*/*w* PEC) or in the presence of both (Panel **B**). All the films were prepared at pH 7.5.

For all the films this weight loss is about 15%–17%. After this, a second and more significant mass fall appears, corresponding to the degradation of the organic films. In particular, for films containing only PEC, degradation started at about 200 °C for both concentrations of the FFS (0.6% and 1%), having a residue of 30% approximately at 400 °C. In contrast with the first step, the second process represents an exothermic transition, as it can be observed in Figure 6. This behavior agrees with previous works [60,61]. As it can be observed in both Panels of Figure 5, the presence of GLY in the films reduces the thermal stability, starting the degradation of the films (at around 170 °C), no matter the presence, or absence of MSNs. An additional sample weight loss was observed at the range of 260–400 °C in PEC film prepared with or without MSNs. In this case, due to the fact that a little difference percentage is observed, the weight loss could be related to the decomposition and pyrolytic de-polymerization of the PEC [62,63]. In films made of either 0.6% or 1.0% of PEC, the presence of MSNs results in a small increment of the residual mass, which can be justified according to the high stability of this inorganic nanomaterial [64].

Figure 6 and Table 2, show thermal properties of PEC-based films. Thermal properties depend mainly on both material chemical composition and state transition occurring during material processing, as well as on the interdependence of both these factors [65]. In Figure 6, it can be observed that, in both types of films (0.6% PEC, Panel A and 1% PEC, Panel B), heat flow alterations in thermal transition occurred between 65 and 100 °C. This behavior is mainly due to the water evaporation, which is an endothermic process. Similar results were obtained by Nisar et al. [66] that studied PEC-based materials as well.

In Table 2 there is the list of DSC thermal parameters such as glass transition temperature (T*g*) and melting temperature (T*m*). T*g* of the 0.6% and 1% PEC is 120.3 °C and 125.8 °C, respectively. A similar result was obtained by Seslija et al. [67], that reported a T*g* equal to 131.0 °C for 0.5% PEC solutions. We observed that the T*g* of the 0.6% PEC film decreased from 120.3 °C to 84.9 °C by adding 30% GLY, while was 91.4 °C when 50% GLY was added to 1% PEC. These were expected results because it is well known that GLY, acting as a plasticizer, reduces the T*g* and also the T*m* [68,69]. MSNs increased the film thermal stability of both kinds of films since the T*g* was 174.4 °C for 0.6% PEC films, and 170.1 °C for 1% PEC films. The addition of GLY gave rise to films with a T*g* equal to 150.0 °C and 146.0 °C, respectively, indicating the thermal protective effect of the plasticizer. Moreover, T*m* of 0.6% and 1% PEC films was at 222.8 °C and 227.4 °C, respectively, and it decreased in PEC+GLY samples, being 205.7 °C and 202.0 °C respectively. The presence of MSN in PEC+GLY+MSN films decreased to 204.8 °C and 200.4 °C, respectively. Our results are in accordance with the previous studies carried out by Nisar et al. [66], that found out that the T*m* of PEC film was 228.9 °C, a value very similar to the one obtained with 1% PEC-based films. 

### 3.6. Quality of Wrapped Strawberries During Storage

Due to their characteristics (mechanical, sealing and permeability properties) 0.6% PEC+30% GLY films containing 3% MSNs (PEC+MSN+GLY) or not (PEC+GLY) were used to wrap strawberries. Experiments were set up using different groups of strawberries wrapped with different films, including Mater-Bi^®^, a commercial bioplastic. The results were compared to unwrapped strawberries (UW). The performed analyses were finalized to study the quality of strawberries from time 0 to time 8 days. 

#### 3.6.1. Weight Loss, pH and Titratable Acidity

**Figure 7:**
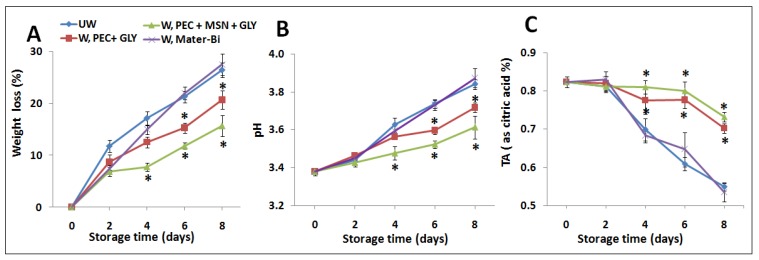
Effect of wrapping with pectin films on weight loss % (**A**), pH (**B**), and titratable acidity (**C**) of strawberries stored for several days (0–8 days). * indicates statistically significant differences at *p* < 0.05 compared to the unwrapping group (UW) used as a control, and to samples wrapped with Mater-Bi^®^.

Figure 7 shows the effect of wrapping on the weight loss, pH and acidity of wrapped fruits with films not containing silica (W, PEC+GLY) and containing silica (W, PEC+MSN+GLY), compared to the effect due to wrapping made with Mater-Bi^®^ (W, Mater-Bi) as commercial material. Unwrapped strawberries (UW) were used as a control. The analyses were performed from 0 to 8 days. Up to two days, no significant differences were found among all the analyzed samples. However, for longer storage times, PEC+GLY and PEC+GLY+MSN films affected positively all the three studied parameters compared to both unwrapped and Mater-Bi^®^ wrapped samples. Thus, as shown in Figure 7A, the weight loss was higher in the latter samples, while it was reduced when strawberries were wrapped with PEC+GLY films or with PEC+MSN+GLY films. The same trend was observed for pH (Figure 7B) that was close to the control values in samples wrapped with both PEC containing films. This is due to the low conversion rate of organic acids during respiration [37,70]. The content of citric acid (Figure 7C) was also higher in both PEC-containing films, even after eight days, compared to the unwrapped samples or the samples wrapped with Mater-Bi^®^. Generally, most fresh fruits are acidic, and the acid content usually decreases during ripening. This is due to the utilization of organic acids during respiration [70]. 

#### 3.6.2. Ascorbic Acid Content and Antioxidant Activity

**Figure 8:**
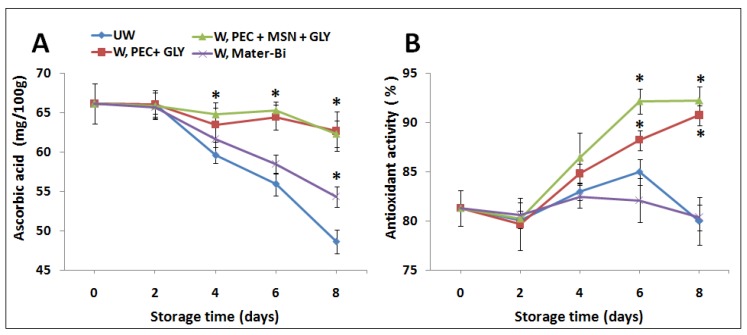
Effect of wrapping with pectin films on ascorbic acid content (**A**), and antioxidant activity (**B**) of strawberries stored for several days (0–8 days). * indicates statistically significant differences at *p* < 0.05 compared to the unwrapping group (UW) used as a control and to samples wrapped with Mater-Bi^®^.

Results shown in Figure 8 indicate a strong reduction of the ascorbic acid in the control UW strawberry and also strawberry wrapped by Mater-Bi^®^ (W, Mater-Bi). Ascorbic acid drop is evident after two day storage. After this time only samples wrapped with both types of PEC-containing films kept high values of ascorbic acid that were only around 5% lower that the control. 

Similar results were obtained by Liu et al. [34], that demonstrated that packaged strawberries with chitosan–poly(vinylalcohol) films reduced the oxidation of ascorbic acid compared to unpackaged ones. Regarding the antioxidant activity, strawberries wrapped by PEC+GLY with or without MSNs showed significantly higher values during the storage times compared to the UW and W, Mater-Bi and to time 0 (Figure 8B). A possible explanation could be related to the higher O_2_ barrier provided by both PEC-based films compared to the barrier property of Mater-Bi (see Table 1). O_2_ concentrations in wrapped samples contrast the evaporation of phenolic compounds that, as it is well known [35,71], are correlated to the antioxidant activity of the fruits. 

#### 3.6.3. Texture Profile Analysis (TPA)

The studied unwrapped and wrapped strawberries were analyzed also to assess their texture profile. Thus, Table 3 shows the TPA (hardness, chewiness and gumminess) of the fruit samples at different storage times (0, 4 and 8 days). 

The results show that, after four days’ long storage, no significant differences were detected on the hardness, chewiness and gumminess between UW strawberries and both W, PEC+GLY and W, PEC+GLY+MSN wrapped fruits. 

**Figure 9:**
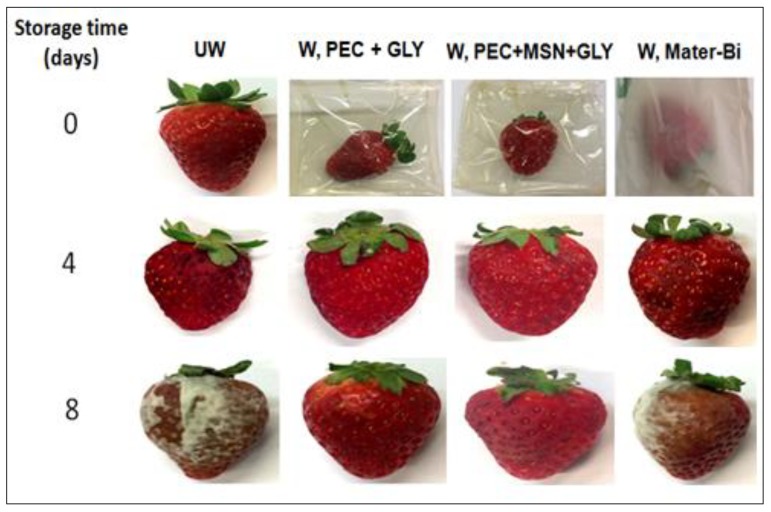
Images of unwrapped and wrapped groups of strawberries during storage time (days).

This means that wrapping is not necessary up to four days to have fresh strawberries. However, results after eight days clearly showed that, while both unwrapped and Mater-Bi^®^-wrapped samples appeared spoiled (Figure 9), the PEC-based wrapping films positively influence the fruit quality. It is interesting to note that W, Mater-Bi samples showed a hardness significantly lower that the UW strawberries, meaning that the fruits were more susceptible to spoiling. These results are consistent with the fact that Mater-Bi^®^ provides a higher barrier to CO_2_ (Table 1), a gas that influences ethylene production inside the wrapping film, thus promoting ripening [72].

## 4. Conclusions

In this paper the effectiveness of MS nanoparticles and GLY on improving the technological features of PEC films was demonstrated. The GLY-enriched films either prepared in the absence or presence of MSNs exploited for strawberry wrapping and different fruit quality parameters were analyzed over a period of eight days. 

The obtained results showed the positive influence of films prepared in the presence of MSNs on fruit weight loss and antioxidant activity, whereas pH, citric and ascorbic acid and texture, seem to be not significantly different from the unwrapped samples. Based on these studies it is possible to suggest PEC-based films prepared in the presence of MSN and GLY as adequate candidates for extending the shelf-life of different fruits.

## Figures and Tables

**Table 1:** Gas and WV permeability of 0.6% and 1% PEC films prepared at pH 7.5 in the presence or in the absence of GLY and/or MSNs.

Film	Permeability (cm^3^ mm m^−2^ day^−1^ kPa^−1^)		
	CO_2_	O_2_	WV
0.6% PEC	0.141 ± 0.009	0.151 ± 0.008	0.159 ± 0.048
+3% MSNs	0.110 ± 0.004 ^a^	0.090 ± 0.014 ^a^	0.083 ± 0.022 ^a^
+30% GLY	0.105 ± 0.012 ^a^	0.037 ± 0.007 ^a^	0.110 ± 0.010
+3% MSNs + 30% GLY	0.076 ± 0.008 ^a,b^	0.022 ± 0.001 ^a,b^	0.026 ± 0.001 ^a,b^
1% PEC	0.081 ± 0.031	0.013 ± 0.001	0.127 ± 0.016
+1% MSNs	0.076 ± 0.001	0.008 ± 0.002 ^a^	0.091 ± 0.010 ^a^
+50% GLY	0.415 ± 0.010 ^a^	0.026 ± 0.012 ^a^	0.122 ± 0.001
+1% MSNs + 50% GLY	0.286 ± 0.006 ^a,b^	0.019 ± 0.003 ^a^	0.085 ± 0.002 ^a,b^
Chitosan (0.6%) *	15.81 ± 2.06	14.33 ± 0.29	0.05 ± 0.02
Viscofan (NDX) **	3.71 ± 0.16	0.03 ± 0.01	0.08 ± 0.01
Mater-Bi^®^ (S-301) **	5.23 ± 0.01	0.74 ± 0.01	0.04 ± 0.01

The values significantly different from those obtained by PEC only (0.6% or 1%) are indicated by “^a^”, while the values indicated by “^b^” were significantly different from those obtained by only adding GLY (*p* < 0.05), * Data from [58] ** Data from [54].

**Table 2:** DSC thermal parameters, glass transition temperature (T*g*) and peak melting temperature (T*m*) of pectin films incorporated with GLY and MSNs, or with both.

Film	T*g* °C	T*m* °C
0.6% PEC	120.3	222.8
+3% MSNs	174.4	224.8
+30% GLY	84.9	205.7
+3% MSNs + 30% GLY	150.4	204.8
1% PEC	125.8	227.4
+1% MSNs	170.1	227.7
+50% GLY	91.4	202.0
+1% MSNs + 50% GLY	146.2	200.4

**Table 3:** Texture Profile Analysis (TPA) of strawberries wrapped or not with 0.6% PEC+30%GLY-based films prepared in the absence (W, PEC+GLY), and the presence of 3% MSNs (W, PEC+MSN+GLY). Unwrapped strawberries (UW) were used as control. A strawberry group was wrapped with Mater-Bi^®^ (W, Mater-Bi). The analyses were performed at 0, 4 and 8 days.

Storage Time (Days)	Strawberry Sample	Hardness (N)	Chewiness (N.mm)	Gumminess (N)
0	UW	57.8 ± 8.9	92.5 ± 10.4	7.9 ± 2.8
4	UW	46.6 ± 5.4	89.2 ± 15.6	7.4 ± 1.9
	W, PEC+GLY	59.9 ± 6.4	81.5 ± 9.6	8.7 ± 2.7
	W, PEC+MSN+GLY	56.1 ± 3.1	74.2 ± 7.6	6.2 ± 1.0
	W, Mater-Bi	43.1 ± 1.3 ^b^	76.7 ± 5.2	6.4 ± 0.4
8	UW	ND	ND	ND
	W, PEC+GLY	34.5 ± 3.8 ^a,b^	50.4 ± 9.3 ^a,b^	4.2 ± 0.8 ^a,b^
	W, PEC+MSN+GLY	25.4 ± 7.3 ^a,b^	41.1 ± 4.8 ^a,b^	3.5 ± 0.4 ^a,b^
	W, Mater-Bi	ND	ND	ND

The values significantly different from those obtained in UW in the same storage time are indicated by “^a^”, while the values indicated by “^b^” were significantly different from those obtained by UW at 0 day (*p* < 0.05). ND: not detected.
